# Disease-specific health spending by age, sex, and type of care in Norway: a national health registry study

**DOI:** 10.1186/s12916-023-02896-6

**Published:** 2023-06-06

**Authors:** Jonas Minet Kinge, Joseph L. Dieleman, Øystein Karlstad, Ann Kristin Knudsen, Søren Toksvig Klitkou, Simon I. Hay, Theo Vos, Christopher J. L. Murray, Stein Emil Vollset

**Affiliations:** 1 Norwegian Institute of Public Health, Postboks 222-Skøyen, 0213 Oslo, Norway; 2 Department of Health Management and Health Economics, Faculty of Medicine, University of Oslo, Oslo, Norway; 3 Institute for Health Metrics and Evaluation, University of Washington, Seattle, WA USA; 4 MSD Norway, Oslo, Norway

**Keywords:** Disease expenditures, Cost of illness, Burden of disease, Disability-adjusted life years (DALYs), Norway

## Abstract

**Background:**

Norway is a high-income nation with universal tax-financed health care and among the highest per person health spending in the world. This study estimates Norwegian health expenditures by health condition, age, and sex, and compares it with disability-adjusted life-years (DALYs).

**Methods:**

Government budgets, reimbursement databases, patient registries, and prescription databases were combined to estimate spending for 144 health conditions, 38 age and sex groups, and eight types of care (GPs; physiotherapists & chiropractors; specialized outpatient; day patient; inpatient; prescription drugs; home-based care; and nursing homes) totaling 174,157,766 encounters. Diagnoses were in accordance with the Global Burden of Disease study (GBD). The spending estimates were adjusted, by redistributing excess spending associated with each comorbidity. Disease-specific DALYs were gathered from GBD 2019.

**Results:**

The top five aggregate causes of Norwegian health spending in 2019 were mental and substance use disorders (20.7%), neurological disorders (15.4%), cardiovascular diseases (10.1%), diabetes, kidney, and urinary diseases (9.0%), and neoplasms (7.2%). Spending increased sharply with age. Among 144 health conditions, dementias had the highest health spending, with 10.2% of total spending, and 78% of this spending was incurred at nursing homes. The second largest was falls estimated at 4.6% of total spending. Spending in those aged 15–49 was dominated by mental and substance use disorders, with 46.0% of total spending. Accounting for longevity, spending per female was greater than spending per male, particularly for musculoskeletal disorders, dementias, and falls. Spending correlated well with DALYs (Correlation *r* = 0.77, 95% CI 0.67–0.87), and the correlation of spending with non-fatal disease burden (*r* = 0.83, 0.76–0.90) was more pronounced than with mortality (*r* = 0.58, 0.43–0.72).

**Conclusions:**

Health spending was high for long-term disabilities in older age groups. Research and development into more effective interventions for the disabling high-cost diseases is urgently needed.

**Supplementary Information:**

The online version contains supplementary material available at 10.1186/s12916-023-02896-6.

## Background

Norway is a high-income nation with a more evenly distributed income and wealth than most OECD countries [1]. The health care system is universal, and primarily tax-funded, with low out-of-pocket spending [2–4]. Health care spending in Norway in 2019 was one of the highest in the world, with more than 6700 USD PPP per capita, corresponding to 10.5% of the Norwegian gross domestic product [2]. Given the high level of spending, it is important to understand in greater detail the age-sex groups and diseases that account for this large expenditure.

Although disease-specific spending estimates are common, variations in concepts, data, and methods often make them incomparable across disease groups and countries [5]. Furthermore, they often include double counting and estimates of how national spending is distributed across diseases are rare [6, 7]. Significant efforts to study health spending have been made in the US, Australia, New Zealand, and Switzerland, though a comprehensive overview of spending by health condition in the Norwegian single-payer (tax-funded) health care system, is lacking [6, 8–10]. Also, those studies that have been done are dated and do not include all components of long-term care.

Little attention has also been paid to the association between burden of disease and health spending across disease categories. Such considerations are relevant given that a high disease burden is often used to justify resources aimed at disease-specific research, and/or garner support for general regulatory or institutional policies to alleviate burden [11]. High health spending may in principle provide further justification for such actions and policies.

The objective of this study was to estimate Norwegian health spending systematically and comprehensively, according to health condition, age and sex group, and type of care, and to explore associations with other health metrics. Some commentary is then made in the discussion on what this means in the near and longer term for Norway and the extent to which these findings may be generalizable to other high-income nations with universal health care.

## Methods

The study was conducted on de-identified health care encounter data from national health registries. The study was approved, and participant consent was waived, by the Regional Committee for Medical and Health Research Ethics South-East Norway, reference number 184544. In addition to government reports and national health accounts, the data came from the following national health registries, covering all public and most of private health care in Norway [12].

### Primary care

The Norwegian Registry for Primary Health Care (NRPHC) is a mandatory register in two parts. The first is based on the National Database for the Reimbursement of Health Expenses (KUHR), which consist of all reimbursement claims sent to the government by primary care physicians, out-of-hour services (i.e., emergency room), private practicing specialists, psychologists, chiropractors, and physiotherapists in Norway [13]. The registry also contains information about the patient (age, sex) and a disease code. The health care providers are required to submit at least one disease code per encounter to be reimbursed. This is done using the International Classification of Primary Care version 2 (ICPC-2) coding system (Additional File 1, Part I) [14, 15]. Physicians are only required to submit one ICPC-2 code to be reimbursed. Though, they can choose to submit multiple codes, and in this case, the first listed diagnosis was considered the primary diagnosis. Private practicing specialist and psychologist submit International Statistical Classification of Diseases and Related Health Problems (ICD-10) codes.

### Home-based care and nursing homes

The second part of NRPHC contains information about home-based services and nursing homes. It is based on the Individual-based Statistics for Nursing and Care Services (IPLOS) registry. This registry forms the basis for mandatory national statistics for municipal care. The registry collects information about each long-term care (LTC) patient or care service recipient in Norway. The registry contains information about persons (age, sex) and their housing situation, assessment by physician/dental services, relevant diagnosis (ICD-10 or ICPC-2), time of application and decision, services received, functional ability, and the care need [12].

### Specialist somatic health care services

The Norwegian Patient Registry (NPR) is a complete population-based nationally administrative health registry, which covers all public outpatient (a patient who comes into the hospital for a short consultation), day patient (a formally admitted patient who comes in for a planned medical service, but discharged the same day), and inpatient (a patient who stays in hospital for one or more nights), in Norway. As the NPR is the basis for funding of hospital and specialist services, it is compulsory to report primary and secondary diagnoses as well as procedures conducted during each contact. The somatic part of NPR contains information about the patient (age, sex), the type of care received, Diagnosis-Related Groups of type of intervention received (DRGs), and up to 21 ICD-10 disease codes for each contact.

### Specialist mental health care services

NPR further contains Mental Health Facilities in Adults; Specialized Interdisciplinary Addiction Treatment; and Mental Health-care Facilities for Children and Youths (BUP). These registries contain information about the patient (age, sex), and the type of care received. Up to 21 disease codes are provided for each admission, using ICD-10. BUP use a modified version of ICD-10 in children, with up to 10 clinical psychiatric disease codes provided.

### Prescribed pharmaceuticals

The Norwegian Prescription Database (NorPD) contains a complete listing of all prescription drugs dispensed by Norwegian pharmacies to non-institutionalized individuals. Reporting is mandatory by Norwegian law. Drugs are classified according to the international Anatomical Therapeutic Chemical (ATC) classification system and reimbursed prescriptions have a reimbursement code for the condition being treated (ICD-10 or ICPC2). The database also contains the persons (age, sex), the drugs (e.g., brand name, strength, package size, and dispensing date), and the pharmacy retail price.

All pharmaceuticals consumed by inpatients, day patients, and outpatients were included in that spending and not in the spending for prescribed pharmaceuticals. Furthermore, the spending for the hospital-financed prescriptions were calculated separately, based on the Norwegian Prescription Database, but were included as hospital spending and not as pharmaceutical spending in accordance with the National Health Accounts.

### Burden of disease

Data on DALYs by health condition in 2019 are from the Global Burden of Disease Collaborative Network [16]. Years of life lost (YLLs) are the product of the number of deaths, by age, sex, and health condition, multiplied by the reference life expectancy for that age. Reference life expectancy is based on the lowest observed risk for death for each age category across all populations with more than five million people. Years lived with disability (YLDs) are calculated from prevalence, distribution of sequelae representing levels of severity or disease consequences, and a disability weight for each sequela. DALYs are the sum of the two components YLL and YLD, and are used as a summary measure of health in a given year [17].

### Estimation strategy

The project used microdata with national coverage to estimate health spending for 144 conditions, 38 groups for age and sex, 8 types of care (general practitioners; physiotherapists and chiropractors; specialized outpatient; day patient; inpatient; prescription drugs; home-based long-term care; and nursing homes) in Norway in 2019. The 38 groups for age and sex used in this study included 19 age groups for each sex, including younger than 1 year, 1 to 4 years, 5 to 9 years, … 85 and above. The 144 health conditions are presented as 14 aggregated conditions (Additional File 1, Supplementary Table 1) and are developed by the Institute for Health Metrics and Evaluations for the Disease Expenditure Project (DEX) [8].

The process used to generate spending estimates was based on a theoretical framework of the DEX-project [8]. The process can be divided into four steps. First, we ensured that each encounter included information about age, sex, and at least one diagnosis code and calculated the cost of each encounter, using prices, claims, and DRGs in the data, and/or duration of encounter combined with unit costs from external sources. More details about the unit costs, estimation methods, and sources can be found in Additional File 1, Part II, and Supplementary Table 2 [13, 18–24]. Second, we assigned a health condition category to each encounter based on disease codes (ICD10 or ICPC-2). Third, as outlined below, we adjusted for data gaps, imperfections, and comorbidities. Fourth, following the cost of illness estimation methods we scaled the estimated spending from the microdata to reflect the official Norwegian spending reported in the Norwegian National Health Accounts [8, 9, 12, 25]. The fourth step is described in Additional file 1, Part III, and Supplemental Table 4 [8, 9, 12, 20, 25, 26].

We calculated Spearman’s rank correlation coefficients between spending and each disease burden metric and CIs using bootstrapping with 1000 draws.

#### Adjusting for comorbidities

Following Dieleman and colleagues [8] spending estimates in specialist care, home-based care, and nursing home care were adjusted for comorbidities. To redistribute a portion of the spending initially attributed to a primary diagnosis to a comorbid condition, the excess spending associated with each comorbidity was measured using log-linear regression model. Spending associated with a visit or stay for a single health condition was regressed on binary indicators identifying the diagnosis of any other health condition. To avoid spurious associations caused by small sample sizes and outlying data points, lasso regressions were used, which constrained the coefficient estimates for regressions that have many parameters but few observations. Comorbidity adjustment factors, which were used to adjust spending, were then calculated based on the regression coefficients [8].

For specialist care, this was completed separately for 4 age groups (< 1–19 years, 20–44 years, 45–64 years, and ≥ 65 years) and for each health condition that had at least 500 observations for that age group. Due to sparseness, home-based and nursing home care excluded individuals below the age of 65 from the analyses.

#### Addressing data gaps and adjusting for imperfect data

Some encounters were registered using ICPC-2. These ICPC-2 codes were translated into ICD-10 codes using available maps and further categorized to the health conditions [15].

Not all ICD-10 codes fit within one of the 144 health conditions. In cases where a code did not map to a health condition in our cause typology, health conditions were assigned in three steps. First, a health condition was assigned based on the second or third diagnosis, if available. Second, if not available, we replaced the missing health condition with a random draw of observed values from a donor pool of encounters, which were similar with respect to age, sex, type of care, and the first chapter letter in the ICD-10 codes. Third, when primary ICD codes were from the Chapter XVIII “Symptoms, signs” or Chapter XXI “Factors influencing health status and contact with health services” and did not map directly to any of the 144 health conditions, we based the donor pools on age, sex, and type of care. In both steps two and three, age groups were aggregated in cases where donor pools had a low number of observations.

The home-based care and nursing home information required adjustments. First, it contained encounters with missing diagnoses. Second, primary diagnoses were not identified in the data. To assign a primary diagnosis, when multiple diagnoses were listed for an encounter, we first ranked all the health conditions according to nurse-measured individual care needs. We then assigned, to each encounter, the diagnosis associated with the highest care need as the primary diagnosis. When information about diagnosis was completely missing from an encounter a health condition was replaced by a health condition from a donor-pool of encounters with non-missing diagnosis, based on age, sex, and type of care (more on each of these steps are in Additional file 1, Part V).

A disease code was not available for 16.9% of prescription drug spending, i.e., the non-reimbursed prescriptions. Hence, ICD-10 codes were assigned to non-reimbursed prescriptions based on ATC codes. To match ATC codes with ICD-10 codes we used an ATC-to-ICD-10 map developed based on Austrian data [27, 28]. As ATC to ICD-10 maps are specific to the setting, published literature was used to distribute antibiotics and sleep medication and a physician distributed the pain medication [29–31]. Finally, a pharmacoepidemiologist revised the mapping, focusing on the most expensive spending categories.

The reimbursed prescriptions were coded with a modified ICPC2 or ICD-10, which includes some special codes for reimbursement purposes. These special codes were translated into health conditions by a physician.

In this framework, physical injury categories are based on the external cause of injuries in the ICD-10, while, for most encounters, only the nature of injury codes is reported in the health registries. To address this issue, we assigned codes of external causes probabilistically to the nature of injury codes. Probabilities for this re-assignment were generated, by age and sex, from NPR data that included both external cause codes and nature of injury codes, for some contacts.

## Results

These Norwegian national data for 2019 cover 33,785,734 general practitioners contacts; 9,120,680 physiotherapists and chiropractors visits; 13,438,308 specialized outpatient visits; 361,552 day patient stays; 5,005,349 inpatient bed-days; 53,971,524 prescribed pharmaceuticals; 42,402,277 home-based long-term care hours of care received; and 16,072,342 nursing homes bed-days. After scaling the study reflected 83.6% of health care spending in the Norwegian national health accounts in 2019. Not included in this study were dental health care (4.8% of total health care spending); over-the-counter drugs (4.7%); providers of preventive care (2.2%), health system administration (1.5%), and health care in other industries (3.1%).

### Health care spending by health conditions and type of care

**Fig. 1:**
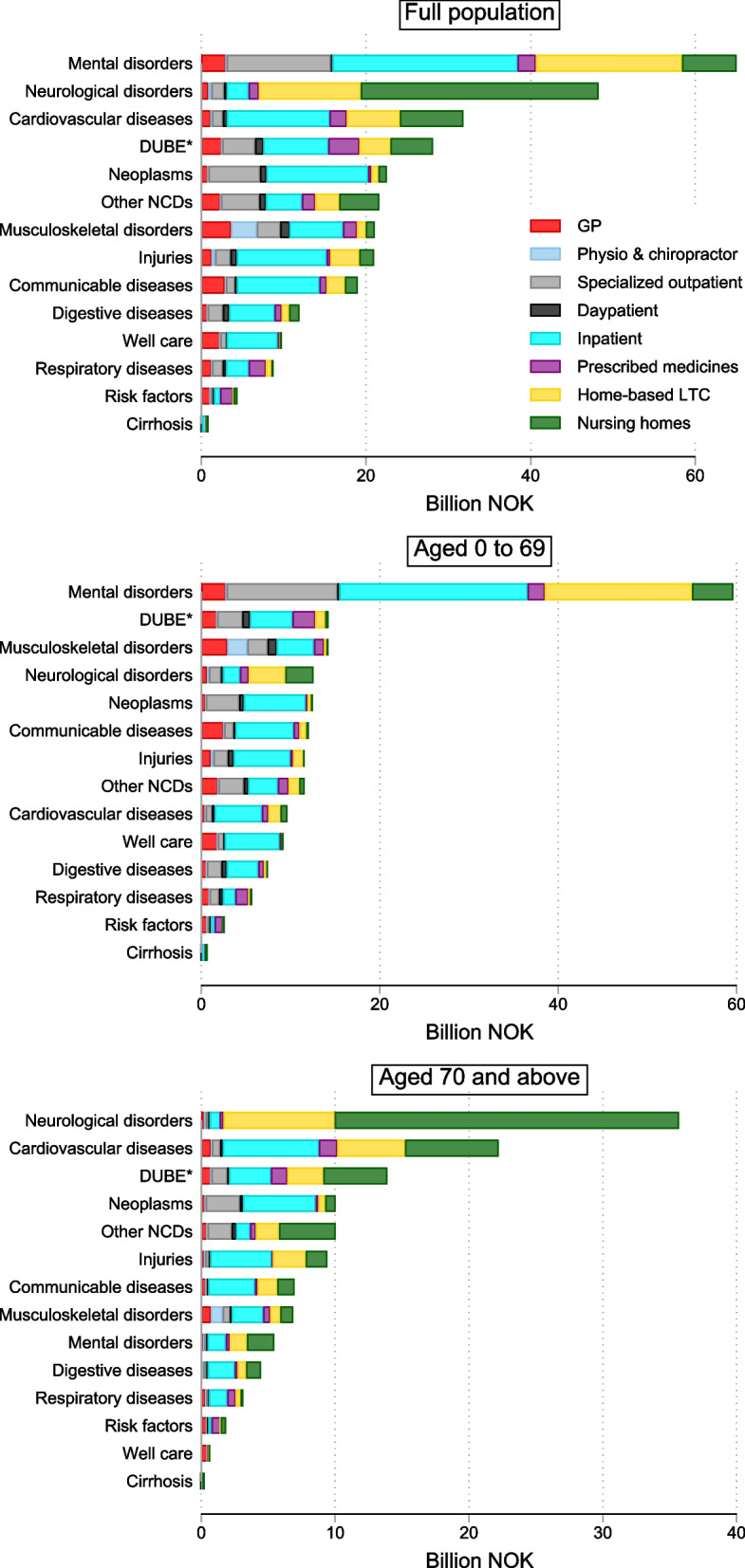
Health care spending in Norway by type of care and health condition category, 2019 *Notes*: * Communicable, maternal, neonatal, and nutritional diseases. ** DUBE indicates diabetes, urogenital, blood, and endocrine diseases. Reported in 2019 Norwegian Kroner. Increases in spending along the *x*-axis show more spending. Additional File 1, Supplementary Table 1 lists the aggregated condition category in which each condition was classified

Among the 14 aggregated conditions, the greatest spending was for mental and substance use disorders: 65.03 billion Norwegian Kroner (NOK) or 20.7% of total spending (Table 1; Fig. 1). The largest categories of mental care spending were for schizophrenia; depressive disorders and anxiety; alcohol and drug use disorders; and intellectual disabilities (due to home-based care) (Table 2; Additional file 1, Supplemental Fig. 1). Neurological disorder, which includes dementias, constitutes 15.4% of total spending, and was the second largest category.

**Table 1:** Norway 2019: Estimated health care spending in 14 aggregated health condition groups by age and type of care

**Aggregated health category**	**Health care spending, 2019 NOK billion**		**Percent by age group (% by columns)**					**Percent by type of care (% by columns)**							
		**% of total**	**< 1**	**1–14**	**15–49**	**50–74**	** ≥ 75**	**GPs**	**Physio. Chiro**	**Day patient**	**Specialized outpatient**	**Inpatient**	**Prescript. drugs**	**Home-based LTC**	**Nursing homes**
Mental and substance use disorders	65.03	20.7	1.4	36.7	45.8	15.5	3.2	12.6	1.1	3.0	31.2	21.3	11.6	33.6	10.9
Neurological disorders	48.24	15.4	0.6	5.0	4.8	11.9	30.1	3.5	8.3	4.0	3.9	2.5	6.5	23.4	47.8
Cardiovascular diseases	31.82	10.1	0.3	0.5	2.0	11.8	17.4	4.7	3.6	7.1	3.3	11.8	10.9	12.2	12.7
Diabetes, urogenital, blood, and endocrine diseases	28.14	9.0	2.1	5.9	6.7	10.0	10.7	10.6	0.7	13.9	9.9	7.5	19.9	7.1	8.5
Neoplasms	22.54	7.2	0.4	3.1	3.5	12.4	6.1	3.4	1.1	11.0	15.5	11.7	2.0	1.7	1.6
Other non-communicable diseases	21.61	6.9	9.8	14.2	5.3	5.8	8.1	9.8	1.7	11.5	11.4	4.2	8.5	5.5	8.0
Musculoskeletal disorders	21.04	6.7	0.4	3.6	6.7	9.7	4.6	15.1	68.1	16.4	7.0	6.2	8.8	2.1	1.7
Injuries	20.98	6.7	1.3	8.6	5.8	6.8	7.4	5.3	9.3	11.2	4.6	10.3	2.2	6.7	2.9
Communicable, maternal, neonatal, and nutritional disorders	18.99	6.0	38.9	9.8	5.5	4.8	5.3	11.9	3.0	3.8	2.7	9.5	4.5	4.2	2.5
Digestive diseases	11.90	3.8	1.1	3.6	4.1	4.2	3.3	3.3	0.1	10.2	4.6	5.3	4.5	1.7	2.0
Well care	9.73	3.1	42.7	2.6	6.4	0.8	0.4	9.7	0.7	1.0	1.7	5.9	1.3	0.2	0.1
Chronic respiratory diseases	8.75	2.8	0.7	5.8	2.4	3.6	1.9	5.2	2.2	6.2	3.1	2.6	10.9	1.2	0.4
Expenditure on risk factors	4.37	1.4	0.0	0.3	0.9	2.2	1.2	4.7	0.2	0.4	1.0	0.7	8.4	0.1	0.7
Cirrhosis of the liver	0.76	0.2	0.1	0.3	0.2	0.4	0.1	0.1	0.0	0.2	0.1	0.5	0.2	0.1	0.0
Total %		100	100	100	100	100	100	100	100	100	100	100	100	100	100
*Total Health Care Spending,2019 NOK Billion*	*313.9*		*5.8*	*14.8*	*88.3*	*101.8*	*103.2*	*24.0*	*4.6*	*6.9*	*40.6*	*105.5*	*19.0*	*53.1*	*61.4*

**Table 2:** Health care estimated spending for the 100 most expensive health conditions of the 144 health conditions analyzed

**Rank (high to low)**	**Health category**	**Health care spending, 2019 NOK billion**	**Type of care (% of health care spending, by rows)**							
			**GPs**	**Phys. Chiro.**	**Day patient**	**Out-patient**	**Inpatient**	**Drugs**	**Home-based LTC**	**Nursing homes**
	*Total*	*313.9*	7.6	1.5	2.2	12.9	33.6	6.0	16.9	19.2
1	Dementias	31.92	0.4	<0.1	<0.1	0.3	1.3	0.2	20.1	77.7
2	Falls	14.52	4.9	1.9	3.2	7.4	51.7	1.2	19.2	10.5
3	Intellectual disability	14.42	0.4	0.1	<0.1	0.8	0.9	<0.1	75.1	22.7
4	Cerebrovascular disease	14.28	1.1	1.0	0.3	0.9	20.0	0.9	32.4	43.4
5	Schizophrenia	10.20	1.3	<0.1	0.3	9.1	46.6	2.7	31.0	9.1
6	Anxiety disorders	9.56	8.8	0.1	0.4	43.4	35.5	3.4	5.9	2.5
7	Sense organ diseases	9.34	8.1	0.2	6.1	32.6	10.8	7.1	10.6	24.6
8	Diabetes mellitus	8.72	8.8	<0.1	0.2	7.1	15.0	18.1	22.7	28.2
9	Skin and subcutaneous	8.64	17.1	0.2	1.3	13.8	23.0	10.5	10.9	23.2
10	Depressive disorders	8.47	10.1	<0.1	0.2	25.2	41.2	6.3	7.3	9.6
11	Other musculoskeletal	8.03	24.3	14.5	10.6	18.0	18.3	5.0	6.1	3.2
12	Urinary diseases/male infertility	6.48	11.9	0.2	3.4	7.2	44.2	13.6	12.4	7.2
13	Low back and neck pain	6.35	19.1	19.9	2.1	10.6	29.6	11.7	4.7	2.4
14	Lower respiratory tract infect.	6.13	5.4	<0.1	0.4	0.7	69.9	3.4	17.1	3.0
15	Drug use disorders	5.41	3.1	<0.1	0.5	15.1	68.3	2.7	7.9	2.5
16	Osteoarthritis	4.79	5.3	12.1	2.5	7.0	56.9	5.1	2.9	8.2
17	Endocrine/metab./blood/imm.	4.48	9.2	0.2	1.9	14.4	34.5	13.1	7.3	19.4
18	Gynecological diseases	4.48	11.7	0.1	13.3	14.4	17.7	8.0	12.9	21.9
19	Other digestive diseases	4.42	13.1	0.1	5.2	15.6	34.5	8.8	12.5	10.2
20	Ischemic heart disease	4.32	5.0	0.2	3.6	7.0	72.9	6.7	2.2	2.3
21	Epilepsy	4.28	1.9	0.3	0.2	3.1	10.5	11.3	43.2	29.4
22	Other unintentional injuries	4.27	8.5	2.0	5.2	14.1	53.8	2.2	10.6	3.5
23	Heart Failure	4.20	3.6	0.1	0.7	3.5	36.7	4.6	32.7	18.1
24	Parkinson's disease	4.14	1.4	2.0	0.2	2.0	5.5	5.0	34.8	49.1
25	Pregnancy and postpart. care	4.10	7.4	<0.1	0.1	5.7	86.5	0.2	<0.1	<0.1
26	Alcohol use disorders	3.93	3.1	<0.1	1.0	10.2	45.2	0.6	23.0	16.8
27	Bipolar disorder	3.64	3.9	<0.1	0.2	16.4	55.7	3.8	16.0	3.9
28	Multiple sclerosis	3.61	1.7	3.6	1.5	17.0	19.7	1.7	41.1	13.6
29	Atrial fibrillation and flutter	3.54	11.4	<0.1	2.1	6.4	34.7	33.4	6.3	5.6
30	Other chronic respiratory	3.35	19.1	0.2	11.9	23.0	21.5	17.4	6.1	0.8
31	Chronic kidney diseases	3.35	1.5	<0.1	0.8	47.8	28.6	8.5	2.1	10.6
32	Colon and rectum cancers	3.25	1.9	<0.1	0.9	23.3	68.4	0.4	2.9	2.1
33	COPD	3.23	9.4	2.0	0.4	4.4	50.3	18.5	11.4	3.6
34	Other cardiovascular	3.06	2.6	<0.1	5.0	11.2	60.3	7.6	3.5	9.7
35	Other neurological disorders	3.02	4.1	2.0	6.3	14.9	23.6	0.8	39.7	8.6
36	Congenital anomalies	2.93	2.1	1.6	2.4	8.7	36.2	0.8	30.7	17.6
37	ADHD	2.83	6.8	0.1	0.4	50.5	16.8	19.2	4.4	1.8
38	Treatment of hypertension	2.78	32.9	<0.1	0.5	5.3	14.2	33.5	0.7	12.8
39	Other mental	2.67	16.7	0.1	0.5	27.2	39.2	7.0	6.9	2.4
40	Breast cancer	2.51	3.0	0.9	5.7	43.4	29.8	4.3	3.1	9.9
41	Well baby	2.39	<0.1	<0.1	<0.1	<0.1	100.0	<0.1	<0.1	<0.1
42	Trachea, bronc., & lung cancers	2.11	2.1	0.2	0.9	30.9	53.2	0.5	5.3	7.0
43	Well person	1.94	75.7	0.6	0.7	12.6	6.5	<0.1	2.4	1.5
44	Inflammatory bowel disease	1.93	5.7	0.1	0.8	47.8	25.8	14.5	1.9	3.4
45	Other neoplasms	1.87	4.6	0.3	6.6	24.0	58.2	0.9	3.7	1.5
46	Eating disorders	1.83	1.3	<0.1	0.2	31.7	62.1	0.2	4.1	0.3
47	Asthma	1.76	16.6	1.9	0.4	15.8	11.7	48.4	2.4	2.7
48	Upper respiratory tract infect,	1.74	69.8	<0.1	0.9	2.2	15.4	11.6	0.1	<0.1
49	Other infectious diseases	1.69	16.9	6.1	0.5	2.4	41.8	3.9	9.5	18.9
50	Gallbladder and biliary	1.69	2.7	<0.1	9.7	3.7	74.4	1.5	2.1	5.9
51	Prostate cancer	1.67	6.3	0.1	1.2	30.5	44.1	10.3	4.2	3.3
52	Rheumatoid arthritis	1.61	9.8	8.6	1.6	22.0	22.6	12.9	8.8	13.7
53	Autistic spectrum disorders	1.59	<0.1	<0.1	0.9	38.5	25.6	<0.1	18.1	16.9
54	Transport injuries	1.38	7.6	2.5	4.1	9.7	55.0	3.7	13.2	4.2
55	Brain and nerv. cancers	1.26	2.2	0.5	0.5	9.6	53.6	0.3	16.1	17.2
56	Leukemia	1.24	1.3	0.1	2.0	18.7	72.8	0.5	4.0	0.7
57	Multiple myeloma	1.19	<0.1	<0.1	3.0	39.4	54.7	0.1	2.1	0.7
58	Migraine	1.16	32.0	5.6	0.6	15.0	11.2	32.5	2.3	0.9
59	Preterm birth complications	1.00	0.1	0.2	0.4	0.4	98.5	<0.1	<0.1	0.5
60	Non-melanoma skin cancer	0.97	31.9	0.2	10.5	37.6	15.5	0.7	2.3	1.3
61	Inguinal or femoral hernia	0.96	2.5	<0.1	28.4	8.7	26.3	<0.1	5.9	28.1
62	Peripheral vascular disease	0.95	3.6	0.2	1.5	8.9	68.3	2.5	3.9	11.2
63	Iron-deficiency anemia	0.94	14.8	<0.1	1.1	6.6	50.3	5.7	16.6	5.0
64	Treatment of hyperlipidemia	0.91	14.0	<0.1	0.7	3.9	5.9	73.6	0.5	1.4
65	Other maternal disorders	0.89	37.3	2.5	2.2	30.3	25.8	0.4	0.5	0.9
66	Non-Hodgkin lymphoma	0.88	2.0	0.3	1.3	26.8	65.0	0.4	2.5	1.8
67	Other nutritional deficiencies	0.84	20.3	<0.1	0.2	1.3	6.3	22.0	15.2	34.7
68	Self-harm and interpers.	0.80	11.1	3.7	3.6	7.3	42.6	11.3	17.1	3.2
69	Peptic ulcer disease	0.79	1.3	<0.1	0.2	2.5	36.7	7.9	23.7	27.8
70	Cirrhosis of the liver	0.76	3.8	0.1	1.5	7.7	68.7	4.1	10.4	3.7
71	Protein-energy malnutrition	0.74	<0.1	<0.1	0.3	3.7	20.4	<0.1	41.4	34.2
72	Aortic aneurysm	0.70	<0.1	<0.1	2.1	8.8	87.2	<0.1	0.9	1.0
73	Oral disorders	0.70	8.5	<0.1	4.9	22.2	46.1	3.7	11.8	2.6
74	Other neonatal disorders	0.69	0.5	<0.1	1.4	2.1	90.4	0.2	3.0	2.3
75	Counselling services	0.69	53.8	2.8	0.3	19.0	8.6	0.3	8.0	7.2
76	Treatment of obesity	0.68	13.5	0.9	0.8	31.9	38.7	<0.1	7.8	6.4
77	Malignant skin melanoma	0.65	<0.1	<0.1	3.6	49.4	40.2	0.9	1.6	4.2
78	Paralytic ileus; intest. obstruct.	0.65	<0.1	<0.1	0.5	0.5	95.1	<0.1	2.4	1.4
79	Bladder cancer	0.64	1.7	<0.1	5.9	28.1	59.9	0.6	2.1	1.6
80	Kidney cancer	0.61	1.9	<0.1	1.8	13.5	77.4	0.4	3.1	1.9
81	Pancreatic cancer	0.60	2.3	<0.1	0.8	16.1	70.5	0.6	5.9	3.7
82	Septicemia	0.58	<0.1	<0.1	1.0	0.1	69.3	0.2	14.4	15.0
83	Appendicitis	0.58	1.6	<0.1	1.5	0.3	92.0	0.1	0.6	4.0
84	Diarrheal diseases	0.55	18.2	<0.1	1.5	3.9	65.6	5.1	1.6	4.1
85	Family planning	0.54	33.1	<0.1	9.4	15.2	0.2	42.1	<0.1	<0.1
86	Hepatitis	0.52	1.6	<0.1	1.7	14.6	26.3	3.8	39.2	12.7
87	Ovarian cancer	0.51	<0.1	<0.1	4.6	31.3	55.9	0.1	2.3	5.8
88	Pancreatitis	0.51	<0.1	<0.1	0.8	3.2	81.6	0.2	2.7	11.5
89	Conduct disorder	0.49	8.1	3.6	0.4	38.3	33.7	2.9	8.9	4.1
90	Uterine cancer	0.46	2.7	<0.1	23.0	26.8	45.6	0.6	0.7	0.7
91	HIV/AIDS	0.45	0.8	<0.1	5.1	23.7	68.5	<0.1	1.9	<0.1
92	Acute renal failure	0.44	<0.1	<0.1	0.8	0.8	75.0	17.1	4.6	1.6
93	Interstitial lung; pulm. sarc	0.40	<0.1	<0.1	1.7	16.7	49.1	10.3	10.7	11.5
94	Stomach cancer	0.37	1.6	<0.1	0.7	16.4	73.6	0.8	3.5	3.3
95	Mouth cancer	0.31	<0.1	<0.1	1.9	19.5	71.9	0.3	1.1	5.3
96	Cardiomyopathy; myocarditis	0.31	1.7	<0.1	1.6	10.5	84.1	0.4	1.1	0.6
97	Otitis media	0.30	41.5	<0.1	14.9	29.2	12.4	0.6	1.3	<0.1
98	Endocarditis	0.28	<0.1	<0.1	0.4	1.0	98.3	0.2	0.1	<0.1
99	Complications of abortion	0.27	6.0	<0.1	20.9	28.4	42.5	2.1	<0.1	<0.1
100	Gout	0.27	20.6	0.2	0.7	8.9	33.5	25.1	7.0	4.1

Among all 144 conditions, the top 20 accounted for 37.5% of spending and were dominated by hospital inpatient, home-based, and inpatient LTC (Table 2). Most resources were estimated for dementias, which accounted for 31.92 billion NOK (10.2% of total spending), and 24.8 billion of these were from nursing home spending alone. The second largest was falls, followed by intellectual disabilities and cerebrovascular disease. Because cancer was disaggregated into 29 conditions, none were among the top 20 conditions with the highest spending but ranked fifth among the aggregated conditions and accounted for 7.2% of total spending.

### Health care spending by age and sex

**Fig. 2:**
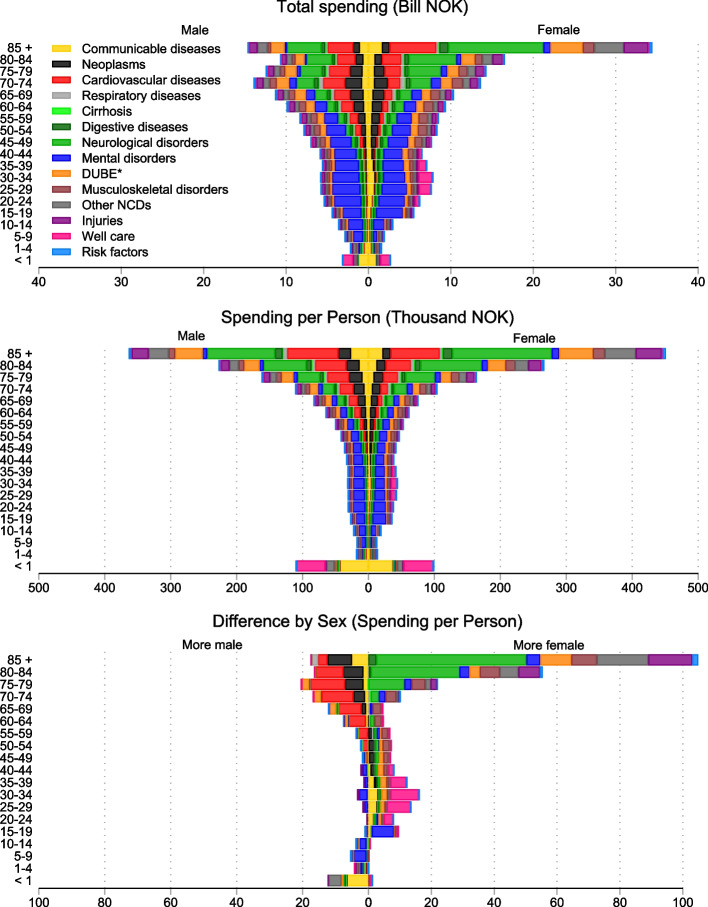
Health care spending in Norway by age, sex, and aggregated condition category, 2019 *Notes*: * Communicable, maternal, neonatal, and nutritional diseases. ** DUBE indicates diabetes, urogenital, blood, and endocrine diseases. Reported in 2019 Norwegian Kroner. Panel **A** illustrates health care spending by age, sex, and aggregated condition category. Panel **B** illustrates health care spending per person. Additional File 1, Supplementary Table 1 lists the aggregated condition category in which each condition was classified. Increases in spending along the *x*-axis show more spending. Population by age and sex is from Statistics Norway and is the 1 of January 2019. Some persons die during the year. Additional analysis accounting for deaths is in Additional File 1, Supplementary Fig. 2

Figure 2 shows that per-person spending increased with age in both men and women, except for neonates and infants younger than 1 year. Spending per person on those younger than 1 year was greater than spending on any other age group younger than 74 years. Those 85 years or older spent more per person on health care than any other age group.

Spending per person was greater among females than males for ages 10 through 59 years and for ages 75 years and older, whereas spending per person was greater among males than females for ages 60 through 74 years and for younger than 10 years (Fig. 2). This could not be explained by premature mortality for males (Additional File 1, Supplementary Fig. 2). For conditions applicable to both sexes, the greatest absolute difference was for CVD followed by neoplasms, for which more was spent on males, and for neurological disorders and injuries, for which more was spent on females. Spending in those aged 15–49 was dominated by mental and substance use disorders, which constituted 46.0% of total spending (Table 1).

### Health care spending and disability-adjusted life years by health condition category

**Fig. 3:**
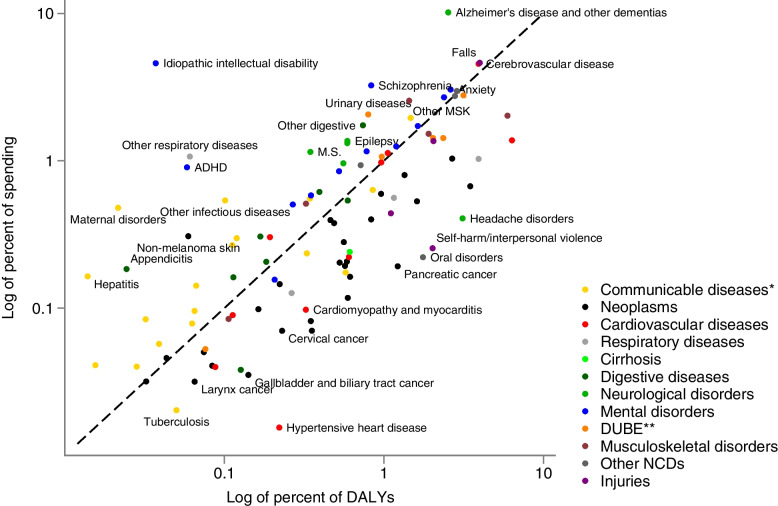
Scatterplot of percent of DALYs and percent of spending in 2019 *Notes*: * Communicable, maternal, neonatal, and nutritional diseases. ** DUBE indicates diabetes, urogenital, blood, and endocrine diseases. Both percent DALYs and percent spending sum to 100%. The solid line represents the line of equality. Data on DALYs by health condition in 2019 are from the Global Burden of Disease Collaborative Network [16]. DEX-health conditions are based upon the GBD health conditions, with a few deviations as health conditions include categories for non-disease-related spending and risk factors. These were excluded. In addition, maternal and neonatal conditions were aggregated. Hence, the scatterplot includes a total of 115 health conditions

Overall, the health conditions with high spending also accounted for a large proportion of DALYs, which resulted in a positive correlation of 0.77 (95% CI 0.67–0.87) (Fig. 3). Dementia was associated with high spending and relatively less DALYs. Ischemic heart disease (IHD) was associated with a large proportion of DALYs, but less spending. The high DALYs from IHD were due to the high mortality for this condition (Additional File 1, Supplementary Fig. 3). In general, spending was more correlated with the YLD (*r* = 0.83, 95% CI 0.76–0.90) component of DALYs, than with the YLL component (*r* = 0.58, 95% CI 0.43–0.72) (Additional File 1, Supplementary Fig. 3).

## Discussion

We estimated Norwegian 2019 health care spending by health condition, age and sex group, and type of care and explored its association with other health metrics, using methods that adjust for comorbidities and data issues. Diseases with a large component of long-term care, like dementia, intellectual disability, and cerebrovascular disease, accounted for the highest amounts of health spending in 2019. Mental disorders like depression, anxiety, and schizophrenia accounted for a large proportion of spending in inpatient and outpatient care, especially in middle-aged individuals. The correlations of numbers of YLD and DALYs with spending were generally high.

### Comparison with related literature

A prior Norwegian study based on data for 2013, showed that 19.5% were spent on mental disorders, 11% on cardiovascular diseases, 10.2% on neoplasms, and 8.9% on musculoskeletal disorders [3]. The prior study did not include LTC. By excluding LTC from the 2019 estimates, the percentages spent on the same categories in the current study were 20.2% for mental disorders, 8.8% on cardiovascular disorders, 10.3% for neoplasms, and 9.4% on musculoskeletal disorders.

By comparing the Norwegian findings, after excluding LTC, with the findings from the US, Switzerland, and New Zealand, we observe that Norway spent much more on mental health care compared with these other nations. This was due to high spending in psychiatric hospitals in Norway [6, 8–10]. Norway also had somewhat lower spending on musculoskeletal disorders, compared with for example US spending. However, the relative difference in spending for cardiovascular diseases and neoplasms was more comparable across the countries.

A large share of Norwegian spending was for LTC; however, few comparable estimates exist for home-based care spending. Still, studies from the US and Australia have included spending in nursing homes and found estimated that 32% and 49% of this spending could be attributed to dementias, respectively [8, 32]. In Norway, 40% of spending in nursing homes was attributed to dementias.

Norway, together with Sweden, Denmark, and the Netherlands, has the highest recorded LTC spending of all OECD countries. These countries also have a high involvement of institutions in LTC and thus little underreporting of LTC spending (see also Additional file 1, Part VI, for a discussion of how these services are recorded in the Norwegian National Health Accounts) [33–36]. Underreporting of LTC, especially for informal care, might be more common in other OECD countries [33].

The disease-specific spending was well aligned with DALYs. This finding supports earlier studies, which have found positive associations between DALYs and spending, but which also suggest that the association between health spending and YLD was more pronounced than for health spending and YLL [3]. Studies from New Zealand and Switzerland showed comparable associations, that emphasize the importance of YLD for predicting health spending [6, 9]. Together, this evidence highlights the cost of caring for individuals with non-fatal health loss. Future analysis would increase policy relevance by comparing the change in DALYs and the change in spending over time jointly to assess the system's overall cost and effectiveness.

### Policy implications

There was a sharp increase in spending by age. Fifteen times as much was spent per female over the age of 85 compared with males aged 25–29, and 38 times as much compared with girls aged 5 to 9. Looking forward, European countries face population aging, which -assuming morbidity by age is static- will result in more long-term illness, and problems in maintaining adequate care within the current health care budgets [37, 38]. A decreasing prevalence of disabling conditions might counteract this demographic effect. Some studies have found a compression of lifetime disabilities [39]. However, not all studies confirm this trend. Musculoskeletal, neurological, and mental disorders, which are large contributors to YLD, show little change over time [16]. Hence, the aging population calls for long-term plans securing a health care system designed to treat the future disease burden of age-related diseases.

A total of 20% of the spending was attributed to mental and substance use, and a high proportion of this spending was caused by conditions with relatively low prevalence. Intellectual disabilities had only 215 prevalent cases per 100,000, but caused high home-based care spending [16]. Comparably, schizophrenia also had only 337 prevalent cases per 100,000, but caused high psychiatric inpatient spending. Anxiety and depressive disorders also had high spending due to inpatient and outpatient psychiatric care, but much higher prevalence per 100,000 of 7124 and 3655 cases, respectively. Thus, spending per case — within mental disorders — varied considerably.

To reduce the health care spending from mental disorders, like anxiety and depression, there is a need for an increased emphasis on prevention. Universal school-based prevention and increased cooperation between specialists and primary care workers have been suggested [40]. Also, by targeting resources on those for whom prevalence reduction can be most readily achieved, resources be freed up to address determinants [40]. Continued research is needed to develop effective prevention and treatments, especially within primary care [40, 41].

We attributed about 40% of nursing home spending to dementia. Four out of five patients in Norwegian nursing homes have dementia and dementia is expected to increase in the near future, both in Norway and globally, as more people live into old age [42, 43]. It is thus critical to prepare and train clinicians and health care workers to identify patients suffering from dementia and provide adequate care. Recent advances underscore the importance of early detection [44]. Prevention studies have highlighted the possibility of targeting risk and protective factors to delay onset, though the effect is modest. No treatment is yet available to halt or reverse the underlying pathology [45]. A continued focus on effective service delivery might also alleviate the pressure on the health service, like the adaption of local communities to reduce the need for extensive home-based health services in those with functional disabilities. Nevertheless, an improved understanding of the disease is needed to develop safe and cost-effective treatments.

### Limitations

This study has several limitations. First, the expenditure estimates do not include costs associated with informal care, which might vary substantially by type of condition and sex. For example, dementias often lead to an extensive burden on, especially female, members of the family [46]. Second, some of the data used in this study was imperfect. 41.4% of home-based and 24.7% of nursing home records had missing diagnoses, which have been imputed based on records with valid diagnoses. It could be an issue that those with longer stays, might be more likely to be recorded with a diagnosis [47]. If this is the case, we overestimated the spending on more severe conditions like dementias in LTC, at the expense of less severe conditions. Third, in primary care, physicians are only required to submit one disease code to be reimbursed. Hence, although the primary diagnosis has been found to correspond well with patient records, comorbidities are likely underreported in the GP data [48] and we have not been able to adjust this part of the analysis for comorbidities. If some health conditions are thought of more often as secondary diseases, and not reported by the GP, the spending on these conditions will be underestimated.

## Conclusions

Spending was generally aligned with disease burden and was higher in females, than males. Spending was substantially higher for older age groups and for long-term disabilities. The aging population calls for long-term plans securing health care funding and a health care system designed to treat the future disease burden of age-related diseases.

### Supplementary Information

**Additional file 1: Supplementary Methods Part I-VI.** Part I: International Classification of Primary Care, Second edition. Part II: Calculation of the cost of encounters. Part III: Scaling estimates to National Health Accounts. Part IV: The cause list. Part V: Addressing data gaps in the homebased care and nursing homes data. Part VI: Home-Based and Institutional Care in the Norwegian National Health Accounts. **Supplementary Table 1.** Aggregated to disaggregated reporting level. **Supplementary Table 2.** Type of care, service unit, source of unit cost and total spending. **Supplementary Table 3.** Percent of total spending on mental disorders by age category. **Supplementary Table 4.** Total spending in the national health accounts and in the microdata, and the scalars. **Supplemental Figure 1.** Mental and Substance Use Care Spending in Norway by Type of Care, 2019. **Supplemental Figure 2.** Health Care Spending per Person, adjusted for deaths in 2019, in Norway by Age, Sex, and Aggregated Condition Category, 2019. **Supplemental Figure 3.** Scatterplot and Correlation of Percent of DALYs, YLDs, YLLs and Percent of Spending in 2019. **Supplemental Figure 4.** Health Care Spending in Norway Type of Care and Age, 2019. **Supplemental Figure 5. **Top 10 Causes of Spending by Type of Care and Sex, 2019.

## Data Availability

The data underlying this article were provided by the Norwegian Directorate of Health and the Norwegian Institute for Public Health by permission. Researchers can gain access to the data by submitting a written application to the data owners at www.helsedata.no.

## Abbreviations

ATC: Anatomical Therapeutic Chemical
BUP: Mental Health-care Facilities for Children and Youths
DALYs: Disability-adjusted life-years
DEX: Disease Expenditure Project
DRGs: Diagnosis-Related Groups of type of intervention received
ICD-10: International Statistical Classification of Diseases and Related Health Problems
ICPC-2: International Classification of Primary Care version 2
IHD: Ischemic heart disease
IPLOS: Individual-based Statistics for Nursing and Care Services
KUHR: The National Database for the Reimbursement of Health Expenses
LTC: Long-term care
NOK: Norwegian Kroner
NorPD: The Norwegian Prescription Database
NPR: The Norwegian Patient Registry
NRPHC: The Norwegian Registry for Primary Health Care
YLDs: Years lived with disability
YLLs: Years of life lost
