# Supplementary material for: Disease-specific health spending by age, sex, and type of care in Norway: a national health registry study
Source: BMC Med. 2023 Jun 6;21:201. doi: 10.1186/s12916-023-02896-6 (PMC10243068; doi:10.1186/s12916-023-02896-6)
Supplement: Supplementary file 1 — Additional file 1: Supplementary Methods Part I-VI. Part I: International Classification of Primary Care, Second edition. Part II: Calculation of the cost of encounters. Part III: Scaling estimates to National Health Accounts. Part IV: The cause list. Part V: Addressing data gaps in the homebased care and nursing homes data. Part VI: Home-Based and Institutional Care in the Norwegian National Health Accounts. Supplementary Table 1. Aggregated to disaggregated reporting level. Supplementary Table 2. Type of care, service unit, source of unit cost and total spending. Supplementary Table 3. Percent of total spending on mental disorders by age category. Supplementary Table 4. Total spending in the national health accounts and in the microdata, and the scalars. Supplemental Figure 1. Mental and Substance Use Care Spending in Norway by Type of Care, 2019. Supplemental Figure 2. Health Care Spending per Person, adjusted for deaths in 2019, in Norway by Age, Sex, and Aggregated Condition Category, 2019. Supplemental Figure 3. Scatterplot and Correlation of Percent of DALYs, YLDs, YLLs and Percent of Spending in 2019. Supplemental Figure 4. Health Care Spending in Norway Type of Care and Age, 2019. Supplemental Figure 5. Top 10 Causes of Spending by Type of Care and Sex, 2019. [file 12916_2023_2896_MOESM1_ESM.pdf]

Additional File1: Supplementary Appendix

Supplements to: Kinge et al. Disease-specific health spending by age, sex, and type of care in Norway: a national health registry study

List of content

Supplementary Methods

Part I: International Classification of Primary Care, Second edition (ICPC-2)
Part II: Calculation of the cost of encounters
Part III: Scaling estimates to National Health Accounts
Part IV: The cause list
Part V: Addressing data gaps in the homebased care and nursing homes data
Part VI: Home-Based and Institutional Care in the Norwegian National Health Accounts

Supplementary Tables

Supplementary Table 1: Aggregated to disaggregated reporting level
Supplementary Table 2: Type of care, service unit, source of unit cost and total spending
Supplementary Table 3: Percent of total spending on mental disorders by age category
Supplementary Table 4: Total spending in the national health accounts and in the microdata, and the scalars

Supplementary Figures

Supplemental Figure 1: Mental and Substance Use Care Spending in Norway by Type of Care, 2019
Supplemental Figure 2: Health Care Spending per Person, adjusted for deaths in 2019, in Norway by Age, Sex, and Aggregated Condition Category, 2019
Supplemental Figure 3: Scatterplot and Correlation of Percent of DALYs, YLDs, YLLs and Percent of Spending in 2019
Supplemental Figure 4: Health Care Spending in Norway Type of Care and Age, 2019
Supplemental Figure 5: Top 10 Causes of Spending by Type of Care and Sex, 2019

References

Supplementary methods

Part I: International Classification of Primary Care, Second edition (ICPC-2)

The ICPC classification was first developed in 1987 by WONCA International Classification Committee, then as HICPIC. The ICPC-2 version, which is the second revision and used in this study, was last updated in March 2003 [1]. The World Health Organization (WHO) has accepted ICPC-2 within the WHO Family of International Classifications. It is used as a classification for primary care or general practice wherever applicable.

ICPC-2 classifies patient data and clinical activity in primary care and allows classification of the patient's reason for encounter, the problems/diagnosis managed, interventions, and the ordering of these data in an episode of care structure [2].

Classification Structure

The ICPC-2 contains 17 chapters: A General and unspecified; B Blood, blood forming organs, lymphatics, spleen; D Digestive; F Eye; H Ear; K Circulatory; L Musculoskeletal; N Neurological; P Psychological; R Respiratory; S Skin; T Endocrine, metabolic and nutritional; U Urology; W Pregnancy, childbirth, family planning; X Female genital system and breast; Y Male genital system; Z Social problems. Each chapter is divided into 7 components dealing with symptoms and complaints (component 1), diagnostic, screening, and preventive procedures (component 2), medication, treatment, and procedures (component 3), test results (component 4), administrative (component 5), referrals and other reasons for encounter (component 6) and diseases (component 7).

Part II: Calculation of the cost of encounters

The following text provide more detail on the calculation of the cost of each encounter in each data source.

Hospital - somatic care

Use of somatic hospital inpatient-, day- and outpatient care episodes were from the Norwegian Patient Register (NPR) for 2019. Costs were calculated from cost-weights using diagnosis-related groups (DRG). In Norway, all somatic hospitals electronically report patient activity to NPR. These reports include information regarding the patient's diagnoses (ICD-10 codes) and the procedures that were used to treat the patient. This information forms the basis for grouping all patient episodes into DRG's which in turn forms the basis for Activity-Based-Funding of Norwegian hospitals. In this way, each patient episode is grouped into one of approximately 900 DRGs, and each DRG holds an associated cost-weight that can be used to estimate the average cost of an inpatient, day- or outpatient care episode. The DRG-costs are designed to cover average costs of a treatment in Norway. Unit cost in 2019 for 1 DRG-point = NOK 51,796 [3].

We use the corrected DRG-points, which is adjusted in accordance with currents regulations. These DRG-points does not include hospital-financed prescriptions, which are drugs prescribed by a hospital physician, dispensed at a hospital pharmacy, but consumed outside the hospital. Spending for these drugs is taken from hospital spending but is calculated separately based on a variable in the prescription drug registry that identifies the financing source. In addition, the corrected DRG-points does not include five types of bundled payments [4]. We include these bundled payments based on the uncorrected DRG-points and the prescription drug registry.

Hospital – psychiatric care

Use of psychiatric hospital inpatient-, day- and outpatient care were identified from NPR in three parts. These were mental health facilities in adults, mental health-care facilities for children and youths and specialized interdisciplinary addiction treatment. Inpatient 2019 psychiatric care was not financed through Activity Based Funding and consequently, we could not use information from DRGs' to estimate these costs. Following GM Bjørnelv, V Halsteinli, BE Kulseng, D Sonntag and RA Ødegaard [5] we use unit cost for hospital inpatient-, day- and outpatient. For inpatient stays, costs were calculated by multiplying the length of stay (in days) by and an estimate of the average cost per day. For day-care, we multiplied each day by half of the cost per day of inpatient care. All outpatient visits were multiplied by an average unit cost estimated based on the length of the consultation.

The Norwegian Directorate of Health do not estimate equal unit costs in the three types of psychiatric care (adults, children and addiction) [6]. Consequently, we apply unique unit costs in each part of the service from 2018. After inflation adjustment, these were NOK 13,110 per inpatient day and NOK 2,721 per outpatient hour, in adults. NOK 19,949 per inpatient night and 3.548 per outpatient hour, in children. NOK 8,075 per inpatient night and NOK 2,721 per outpatient hour, in addiction treatment.

General practitioners

Use of general practitioners (GPs) were identified from the Health Reimbursement Register (KUHR). This register holds information on claims from GPs and patient-copayments [7, 8]. We received information from KUHR on all claims from GP consultations/visits that occurred during 2019, the corresponding reimbursement tariff, and the out-of-pocket payment paid per visit. Following, GM Bjørnelv, V Halsteinli, BE Kulseng, D Sonntag and RA Ødegaard [5] and The Norwegian Directorate of Health [9] we therefore divided the claim and patient-copayment by 0.5 to estimate total costs of care, before scaling to the Norwegian Health Accounts.

Physiotherapist and chiropractors

Use of Physiotherapist and chiropractors were identified from KUHR in the same manner as for primary care physicians.

Private specialist on contract

Use of private specialists on contract (publicly funded) were identified from KUHR in the same manner as for primary care physicians.

Long-term care

Use of long-term care services were identified from the individual-based statistics for nursing and care services (IPLOS) registry. The registry contains information on services provided in LTC, also services classified as social services. The only part of home-based services classified as health services is the type "15 [Health services at home] [10]. Included in institutional LTC are the type of services: "8 [Alleviation - in institution], "17 [Day care in institution]", "22 [Overnight stay in institution]", "21 [Long term stay in institution]", "25 [Municipal emergency help in institution]", "18, 19 & 20 [Time-limited stay]". We do not use a unit cost from an external source, for this part of the service. Instead the unit cost were calculated by dividing total spending from the National Health on 1) the total number of hours in 2019 of face-to-face patient oriented time received for each episode of home-based care, and 2) the total patient time at institution in 2019 for each episode of inpatient LTC.

Prescription drugs

Prescription drugs were identified from the Norwegian Prescription Database (NorPD), which contains a complete listing of all prescription drugs dispensed by Norwegian pharmacies to noninstitutionalized

individuals. The register also contains the pharmacy retail price, which were multiplied with the number of dispensed prescriptions.

Part III: Scaling estimates to National Health Accounts

Spending estimates derived from microdata were scaled to official estimates of yearly health spending published in the National Health Accounts by Statistics Norway. This scaling took place at the aggregated level, for each age, sex, health condition and type of care, in 2019.

Age-, sex-, condition-, and type-specific spending estimates were summed by year and type of care to create yearly spending totals that parallel the National Health Accounts. These totals were then divided by the corresponding National Health Accounts envelopes to generate type of care specific scalars for 2019:

$$Scalar_{TypeOfCare} = \frac{Unscaled\ spending_{TypeOfCare}}{National\ Health\ Accounts\ Envelope_{TypeOfCare}}$$

The scalars used for each type of care are shown in Supplementary Table 4. The scalars with a value below 1 reflect that parts of the expenditures are not included in our prices. Health personnel outside of hospital are paid by a mix of fee-for-service, user charges and capitation. Often the capitation is not included in our microdata. For outpatient treatments, the deductibles are also not included. In addition, laboratory services and radiology, external non-funded services, and capital costs are not included in somatic specialist care microdata. Following the cost-of-illness methodology in prior Norwegian studies, we assume that these expenditures can be distributed in accordance with the explained spending [5, 11-15].

For psychiatric day-care, we multiplied each day by half of the cost per day of psychiatric inpatient care. We observe that the scalar was larger than one, thus this method overestimated the spending, which was then corrected down by scaling the spending.

Part IV: The Cause List

The cause list for this project was based on the Disease Expenditure (DEX) project at the Institute for Health Metrics and Evaluation [11]. Their cause list is in turn based on the Global Burden of Disease Study 2017. GBD 2017 classified causes of health loss at five different levels of disaggregation. The DEX project extracted the Level III classification from GBD 2017. This resulted in a list of 140 causes based on GBD alone. An additional 14 causes were added to the DEX project to account for 4 risk factors and 10 health conditions associated with spending, even if not accounted for by the burden focused GBD project: hypertension, hyperlipidemia, obesity, and tobacco cessation. In addition to these, 10 causes were added that were not associated with health loss but were associated with health spending. Examples of these 10 additional causes were routine health check-ups and uncomplicated labor and delivery, heart failure, septicemia, and renal failure. Spending on these "causes" was tracked because they represent large portions of health spending and are of political interest. A detailed list and map of all causes is in Supplementary Table 1.

Part V: Addressing data gaps in the homebased care and nursing homes data

The homebased care and nursing home information required adjustments for two reasons. First, the dataset did not provide information to identify primary diagnosis, in cases where multiple diagnoses per encounter were listed. Second, some home-based and of nursing home records had missing diagnosis,

which were imputed based on records with valid diagnoses. Below we provide more detail on both adjustments.

Assigning primary diagnosis

Given that an encounter had at least one valid disease code, there were usually multiple diagnoses per encounter. However, the dataset did not record the primary diagnosis leaving us with little knowledge about the main reason for use of homebased care or the reason why the person was in a nursing home. For example, in some cases a diagnosis for dementia would be recorded together with less severe conditions like incontinence and/or asthma.

Following the theory used in the Norwegian Specialist Health Care Services, we wanted to assign the most severe or resource-intensive condition during the episode as the primary diagnosis. To approximate the severity of each condition we used a variable for care need, which were recorded for each patient for each episode by a nurse, using a standardized assessment form. The nurse assesses and records the level of help required for daily housework, food and goods supply, personal hygiene, dressing/undressing, toilet routines, eating, walking around the house, walking around outside, taking care of one's own health, memory, communication, daily life decisions, social activities, controlling one's own behavior, vision, hearing, and whether the applicant receives help from next of kin.[16] The care need information was available to us as a categorical variable with three categories (high, medium, and low).

We regressed the 144 disease categories as dummy variables, on the three categories for care need using an ordered logistic regression, adjusting also for age and sex. Based on each of the 144 coefficients from the ordered logistic regression, we generated a ranking of all health conditions from 1 to 144. We then applied this ranking to each episode and assigned the highest ranked condition as the primary condition. In the example above, dementia would be ranked as more severe compared with incontinence and asthma, and would thus be assigned as the primary condition. This process was conducted separately for homebased care and for nursing home care, to ensure that the ranking was specific to the type of care.

After assigning primary and secondary conditions for each episode, the comorbidity redistribution was conducted, also separately for each step. Hence, incontinence and asthma could still contribute to additional spending, for that episode, however as comorbidities.

Imputing missing diagnosis

To get the age sex distribution, we imputed the records with missing diagnosis, which constituted 41.4% of homebased and 24.7% of nursing home records. The records with missing diagnosis still had valid recordings of age, sex, and type of care. In cases where a disease code was completely missing, we replaced the missing health condition with a random draw of observed values from a donor-pool of encounters, which were similar with respect to age, sex, and type of care.

Part VI: Home-Based and Institutional Care in the Norwegian National Health Accounts

The separation between health and social care has been a challenge in the context of long-term care (LTC) in the health accounts [17]. In theory, the Norwegian Health Accounts should include only health care. In the following, we explain how this has been identified.

In Norway, since 2002, functionally distributed figures from the Municipality-State-Reporting system KOSTRA have been used to indicate the level of total spending for LTC (home-based care and inpatient)

[18, 19]. These figures do not provide a basis for distinguishing health-related from non-health-related services. Therefore, for institutional LTC, figures for places in nursing and retirement homes are used. The proportion of nursing home places is used to distinguish the health-related part of institutional spending, which should be included in the health accounts. In addition to long-term care in institutions, home-based LTC also needs separate out social care from health care. Statistics Norway uses individual-based statistics for nursing and care services (IPLOS) registry to distinguish between health-related and non-health-related care in the home. The proportion of home health services (which includes both somatic and psychiatric care) is used to identify the health-related part of the home-based care expenses, which are included in the Norwegian national health accounts [18, 20].

Supplementary Tables

Supplementary Table 1: Aggregated to disaggregated reporting level

**Aggregate conditions (DEX level 2)**	**Disaggregated conditions (DEX level 3)**
Communicable, maternal, neonatal, and nutritional diseases	Tuberculosis
Communicable, maternal, neonatal, and nutritional diseases	HIV/AIDS
Communicable, maternal, neonatal, and nutritional diseases	Diarrheal diseases
Communicable, maternal, neonatal, and nutritional diseases	Tetanus
Communicable, maternal, neonatal, and nutritional diseases	Measles
Communicable, maternal, neonatal, and nutritional diseases	Varicella
Communicable, maternal, neonatal, and nutritional diseases	Intestinal infectious diseases
Communicable, maternal, neonatal, and nutritional diseases	Lower respiratory tract infections
Communicable, maternal, neonatal, and nutritional diseases	Upper respiratory tract infections
Communicable, maternal, neonatal, and nutritional diseases	Otitis media
Communicable, maternal, neonatal, and nutritional diseases	Meningitis
Communicable, maternal, neonatal, and nutritional diseases	Encephalitis
Communicable, maternal, neonatal, and nutritional diseases	Diphtheria
Communicable, maternal, neonatal, and nutritional diseases	Whooping cough
Communicable, maternal, neonatal, and nutritional diseases	Neglected tropical diseases and malaria
Communicable, maternal, neonatal, and nutritional diseases	Maternal hemorrhage
Communicable, maternal, neonatal, and nutritional diseases	Maternal sepsis and other pregnancy related infection
Communicable, maternal, neonatal, and nutritional diseases	Hypertensive disorders of pregnancy
Communicable, maternal, neonatal, and nutritional diseases	Obstructed labor
Communicable, maternal, neonatal, and nutritional diseases	Complications of abortion
Communicable, maternal, neonatal, and nutritional diseases	Pre-existing medical condition complicating pregnancy or childbirth
Communicable, maternal, neonatal, and nutritional diseases	Other maternal disorders
Communicable, maternal, neonatal, and nutritional diseases	Preterm birth complications
Communicable, maternal, neonatal, and nutritional diseases	Neonatal encephalopathy (birth asphyxia and birth trauma)
Communicable, maternal, neonatal, and nutritional diseases	Sepsis and other infectious disorders of the newborn baby
Communicable, maternal, neonatal, and nutritional diseases	Hemolytic disease in fetus and newborn and other neonatal jaundice
Communicable, maternal, neonatal, and nutritional diseases	Other neonatal disorders
Communicable, maternal, neonatal, and nutritional diseases	Protein-energy malnutrition
Communicable, maternal, neonatal, and nutritional diseases	Iodine deficiency
Communicable, maternal, neonatal, and nutritional diseases	Vitamin A deficiency
Communicable, maternal, neonatal, and nutritional diseases	Iron-deficiency anemia
Communicable, maternal, neonatal, and nutritional diseases	Other nutritional deficiencies
Communicable, maternal, neonatal, and nutritional diseases	Sexually transmitted diseases excluding HIV
Communicable, maternal, neonatal, and nutritional diseases	Hepatitis
Communicable, maternal, neonatal, and nutritional diseases	Leprosy
Communicable, maternal, neonatal, and nutritional diseases	Other infectious diseases
Communicable, maternal, neonatal, and nutritional diseases	Septicemia
Neoplasms	Esophageal cancer
Neoplasms	Colon and rectum cancers
Neoplasms	Mouth cancer
Neoplasms	Nasopharynx cancer
Neoplasms	Other pharynx cancer
Neoplasms	Gallbladder and biliary tract cancer
Neoplasms	Pancreatic cancer
Neoplasms	Malignant skin melanoma
Neoplasms	Non-melanoma skin cancer
Neoplasms	Ovarian cancer
Neoplasms	Testicular cancer
Neoplasms	Stomach cancer
Neoplasms	Kidney cancer
Neoplasms	Bladder cancer
Neoplasms	Brain and nervous system cancers
Neoplasms	Thyroid cancer

Neoplasms	Hodgkin lymphoma
Neoplasms	Non-Hodgkin lymphoma
Neoplasms	Multiple myeloma
Neoplasms	Leukemia
Neoplasms	Other neoplasms
Neoplasms	Liver cancer
Neoplasms	Larynx cancer
Neoplasms	Trachea, bronchus, and lung cancers
Neoplasms	Breast cancer
Neoplasms	Cervical cancer
Neoplasms	Uterine cancer
Neoplasms	Prostate cancer
Other non-communicable diseases	Congenital anomalies
Other non-communicable diseases	Skin and subcutaneous diseases
Other non-communicable diseases	Sense organ diseases
Other non-communicable diseases	Oral disorders
Cardiovascular diseases	Rheumatic heart disease
Cardiovascular diseases	Other cardiovascular and circulatory diseases
Cardiovascular diseases	Heart failure
Cardiovascular diseases	Ischemic heart disease
Cardiovascular diseases	Cerebrovascular disease
Cardiovascular diseases	Hypertensive heart disease
Cardiovascular diseases	Cardiomyopathy and myocarditis
Cardiovascular diseases	Atrial fibrillation and flutter
Cardiovascular diseases	Aortic aneurysm
Cardiovascular diseases	Peripheral vascular disease
Cardiovascular diseases	Endocarditis
Chronic respiratory diseases	Chronic obstructive pulmonary disease
Chronic respiratory diseases	Pneumoconiosis
Chronic respiratory diseases	Asthma
Chronic respiratory diseases	Interstitial lung disease and pulmonary sarcoidosis
Chronic respiratory diseases	Other chronic respiratory diseases
Cirrhosis of the liver	Cirrhosis of the liver
Digestive diseases	Peptic ulcer disease
Digestive diseases	Other digestive diseases
Digestive diseases	Appendicitis
Digestive diseases	Paralytic ileus and intestinal obstruction
Digestive diseases	Inguinal or femoral hernia
Digestive diseases	Inflammatory bowel disease
Digestive diseases	Vascular intestinal disorders
Digestive diseases	Gallbladder and biliary diseases
Digestive diseases	Pancreatitis
Digestive diseases	Gastritis and duodenitis
Neurological disorders	Alzheimer disease and other dementias
Neurological disorders	Parkinson’s disease
Neurological disorders	Epilepsy
Neurological disorders	Multiple sclerosis
Neurological disorders	Migraine
Neurological disorders	Tension-type headache
Neurological disorders	Other neurological disorders
Mental and substance use disorders	Schizophrenia
Mental and substance use disorders	Conduct disorder
Mental and substance use disorders	Idiopathic intellectual disability
Mental and substance use disorders	Other mental and behavioral disorders
Mental and substance use disorders	Alcohol use disorders
Mental and substance use disorders	Drug use disorders
Mental and substance use disorders	Depressive disorders
Mental and substance use disorders	Bipolar disorder
Mental and substance use disorders	Anxiety disorders

Mental and substance use disorders	Eating disorders
Mental and substance use disorders	Autistic spectrum disorders
Mental and substance use disorders	Attention- deficit/hyperactivity disorder
Diabetes, urogenital, blood, and endocrine diseases	Diabetes mellitus
Diabetes, urogenital, blood, and endocrine diseases	Acute glomerulonephritis
Diabetes, urogenital, blood, and endocrine diseases	Chronic kidney diseases
Diabetes, urogenital, blood, and endocrine diseases	Urinary diseases and male infertility
Diabetes, urogenital, blood, and endocrine diseases	Gynecological diseases
Diabetes, urogenital, blood, and endocrine diseases	Hemoglobinopathies and hemolytic anemias
Diabetes, urogenital, blood, and endocrine diseases	Endocrine, metabolic, blood, and immune disorders
Diabetes, urogenital, blood, and endocrine diseases	Acute renal failure
Musculoskeletal disorders	Rheumatoid arthritis
Musculoskeletal disorders	Low back and neck pain
Musculoskeletal disorders	Gout
Musculoskeletal disorders	Osteoarthritis
Musculoskeletal disorders	Other musculoskeletal disorders
Injuries	Transport injuries
Injuries	Falls
Injuries	Other unintentional injuries
Injuries	Self-harm and interpersonal violence
Well care	Well person
Well care	Well baby
Well care	Pregnancy and postpartum care
Well care	Family planning
Well care	Donor
Well care	Counselling services
Well care	Social services
Risk factors	Tobacco intervention
Risk factors	Treatment of obesity
Risk factors	Treatment of hypertension
Risk factors	Treatment of hyperlipidemia

Note: The DEX-cause list is described in more detail in Part V.

Supplementary Table 2: Type of care, service unit, source of unit cost and total spending

TYPE OF CARE	SERVICE UNIT	SOURCE - UNIT COST ESTIMATION	TOTAL SPENDING BY TYPE (MILL NOK)
**Hospital (somatic care)**			113 659
Inpatient care	Stays	DRG-weights* and national DRG unit cost (2019)	
Day-care	Days	DRG-weights* and national DRG unit cost (2019)	
Outpatient care	Visits	DRG-weights* and national DRG unit cost (2019)	
**Mental health hospitals**			33 957
Psychiatric care adults			
Inpatient care	Days	Published unit cost [6]	
Day-care	Days	Published unit cost [6]	
Outpatient care	Visits	Published unit cost [6]	
Mental health-care facilities for children and youths			
Inpatient care	Days	Published unit cost [6]	
Day-care	Days	Published unit cost [6]	
Outpatient care	Visits	Published unit cost [6]	
Drug and alcohol treatment			
Inpatient care	Days	Published unit cost [6]	
Outpatient care	Visits	Published unit cost [6]	
**Private specialist**			5 346
Outpatient care	Visits	Specialist tariff (based on claims) and patient co-payments	
**General practitioner**	Contact **	GP-tariffs (based on claims) and patient co-payments (divided by 0.5 **)	23 962
**Physiotherapist and chiropractors**	Contact	Tariffs based on claims and patient co-payment	4 870
**Long-term care**			
Inpatient (nursing homes)	Days	61 396/total number of days	61 396
Home based care	Hours	53 138/total number of hours	53 138
**Prescription drugs**	Prescriptions dispensed	Pharmacy retail price	18 978

Note: all cost are scaled to match the National Health Accounts as the envelope.[13]

* DRG-weights vary depending on the resources required for a treatment belonging to a specific DRG-category. Unit cost in 2019 for 1 DRG-point = NOK 51,796 EURO 5 257.[3]

** Following, GM Bjørnelv, V Halsteinli, BE Kulseng, D Sonntag and RA Ødegaard [5] and The Norwegian Directorate of Health [9] we therefore divided the claim and patient-copayment by 0.5 to estimate total costs of care, before scaling to the Norwegian Health Accounts.

Supplementary Table 3: Percent of total spending on mental and substance use disorders by age category

	Total	Males	Females
Age<1	1.41	1.69	1.08
Aged 1-14	36.29	38.46	33.35
Aged 15-49	45.97	52.41	40.70
Aged 50-74	15.36	14.81	15.93
Aged 75 & above	3.03	2.70	3.23
All ages	*20.64*	*23.30*	*18.47*

Supplementary Table 4: Total spending in the national health accounts and in the microdata, and the scalars

	National Health Accounts	Sum of spending from microdata	Scalars
**GPs***	23.96	19.95	0.83
**Physio. Chiro.**	4.62	2.52	0.55
**Specialists outside hospital**	5.35	3.87	0.72
**Psychiatric Specialized outpatient**	11.43	9.88	0.86
**Psychiatric Day patient**	0.14	0.18	1.27
**Psychiatric Inpatient**	22.39	20.60	0.92
**Somatic Day patient**	6.41	5.73	0.89
**Somatic Specialized outpatient**	22.67	16.93	0.66
**Somatic Inpatient**	79.04	57.15	0.72
**Prescription drugs**	18.98	14.63	0.77
**Home-based LTC****	53.14	53.14	1
**Institutional LTC*****	60.68	60.68	1

* the sum of microdata spending reflects the claim and patient-copayment divided by 0.5
** the unit cost were generated by dividing the total spending on home-based LTC on the total number of hours of face-to-face time of care received at home.
*** the unit cost were generated by dividing the total spending on Institutional LTC on the total number of hours spent in institution.
The hospital-financed prescriptions are also added to the somatic specialist care services.

Supplementary Figures

Supplemental Figure 1: Mental and Substance Use Care Spending in Norway by Type of Care, 2019

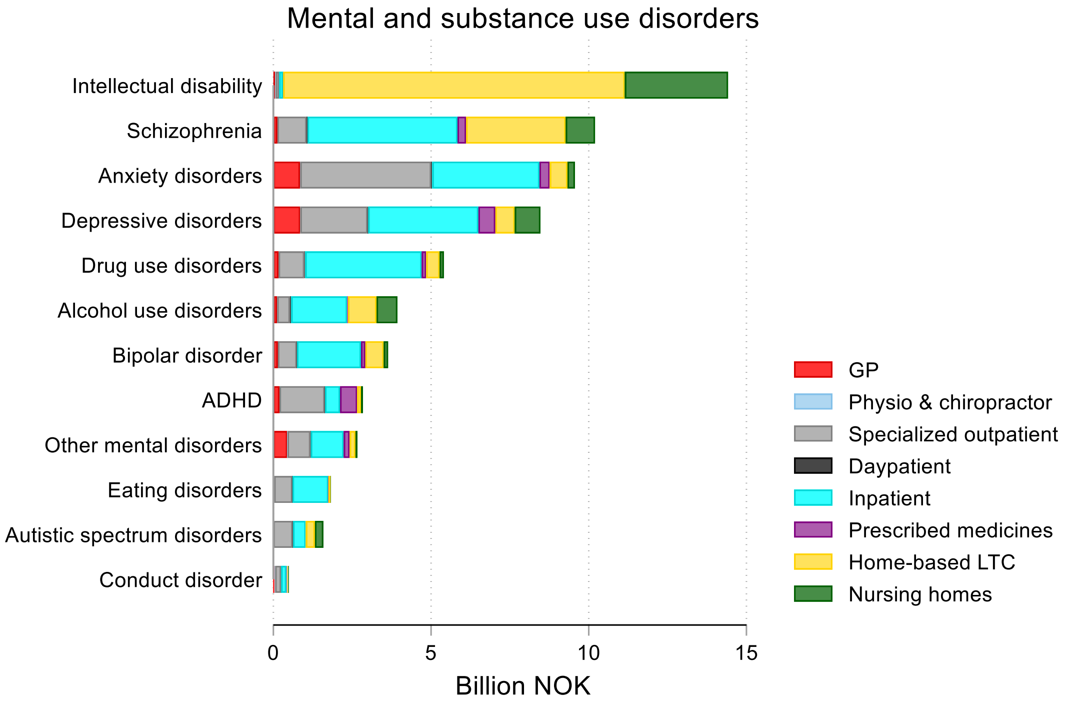

Supplemental Figure 2: Health Care Spending per Person, adjusted for deaths in 2019, in Norway by Age, Sex, and Aggregated Condition Category, 2019

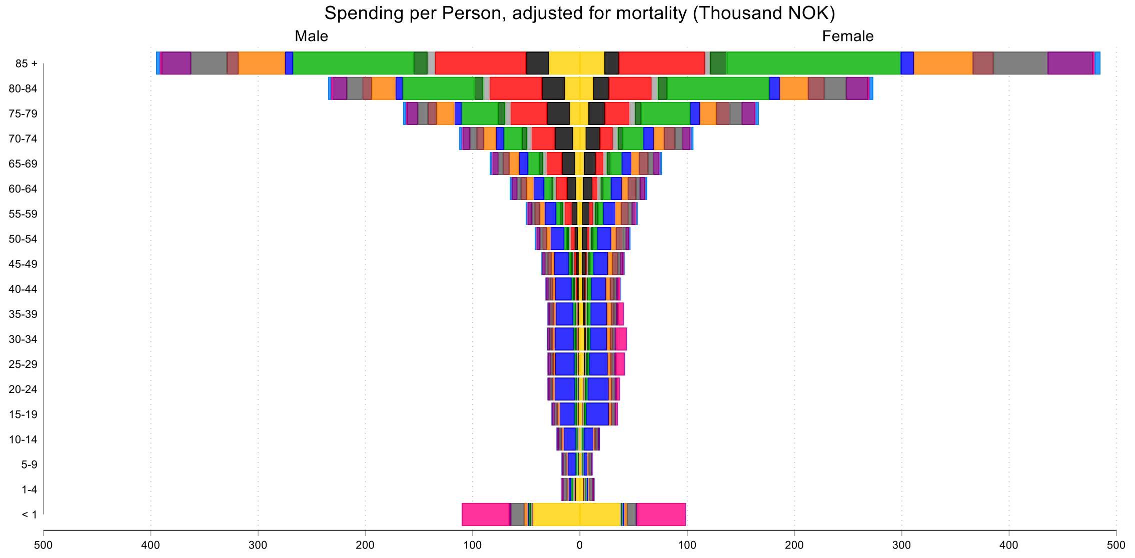

* Communicable, maternal, neonatal, and nutritional diseases
** DUBE indicates diabetes, urogenital, blood, and endocrine diseases.
Reported in 2019 Norwegian Kroner. Panel A illustrates health care spending by age, sex, and aggregated condition category. Panel B illustrates health care spending per person. Supplementary table 3 lists the aggregated condition category in which each condition was classified. Increases in spending along the x-axis show more spending. Population by age and sex is from Statistics Norway and is the 1 of

January 2019. Some persons die during the year, we assume that these live for six months and thus subtract “deaths*0.5” by age and sex from the population count.

Supplemental Figure 3: Scatterplot and Correlation of Percent of DALYs, YLDs, YLLs and Percent of Spending in 2019

A: Spending and DALYs

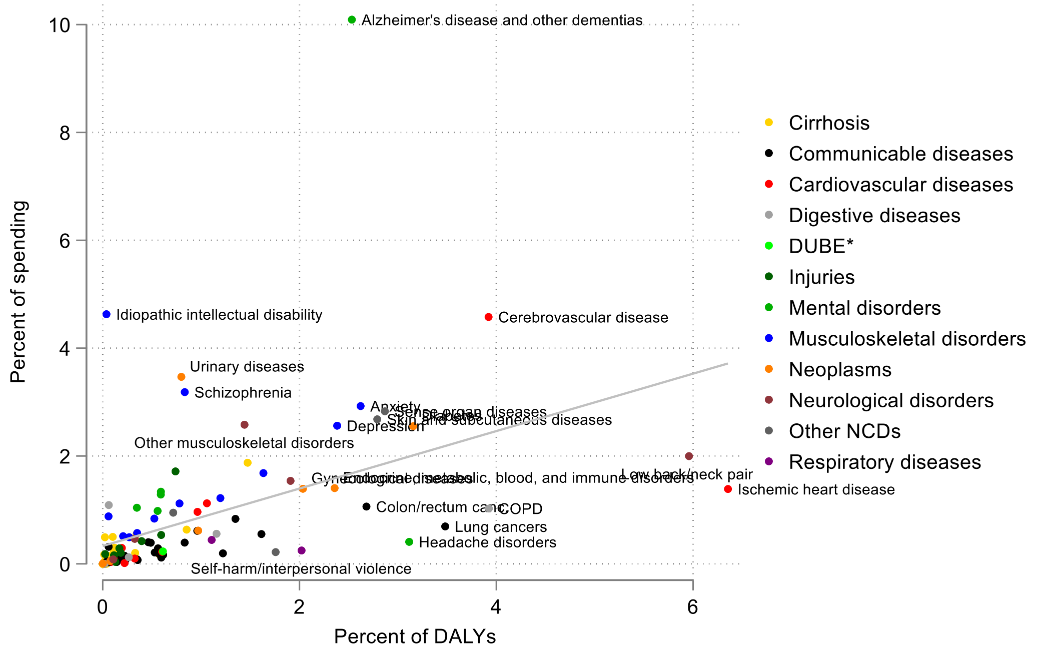

Spearman's correlation coefficient=0.7676

B: Spending and YLDs

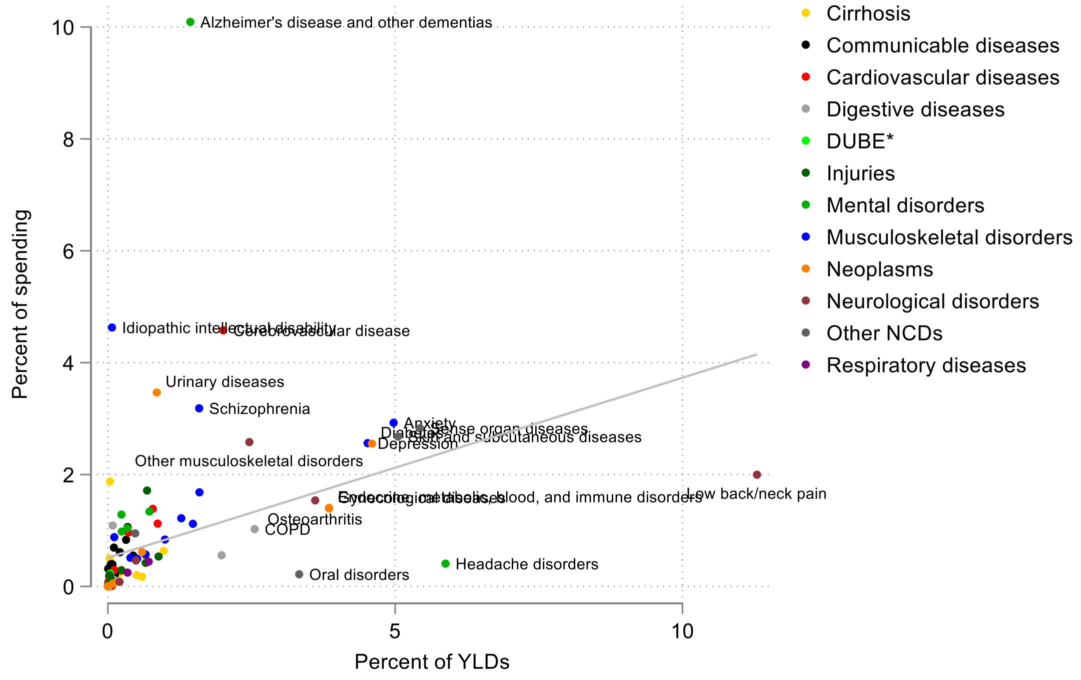

Spearman's correlation coefficient=0.8335

C: Spending and YLLs

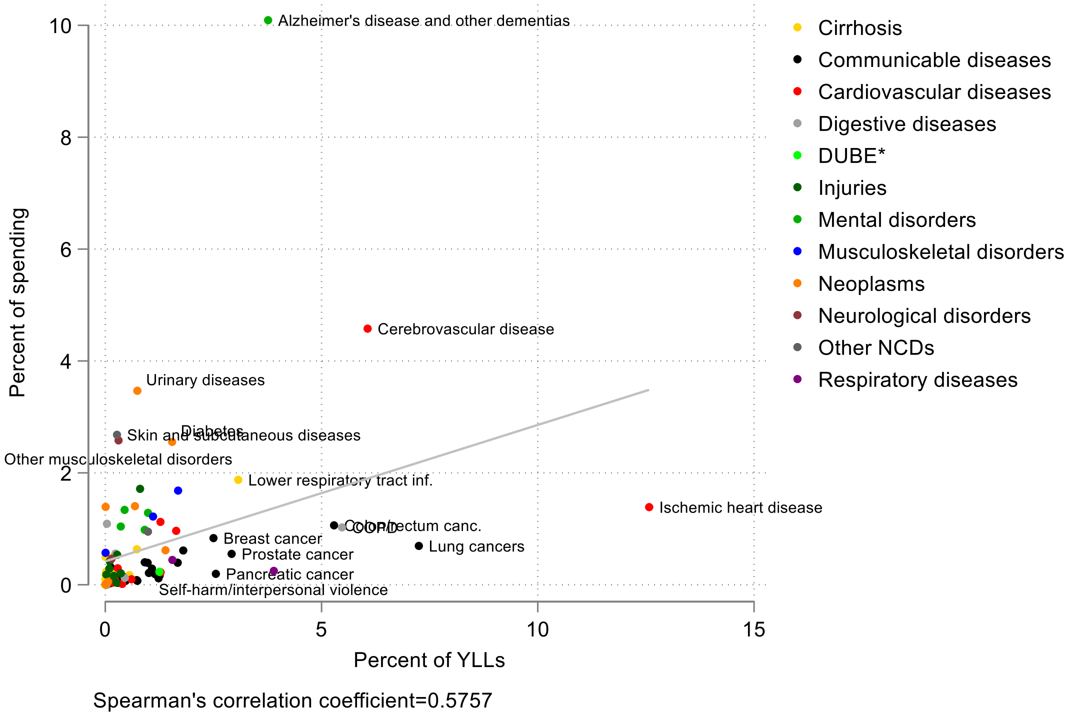

DUBE indicates diabetes, urogenital, blood, and endocrine diseases. Reported in 2019 Norwegian Kroner. Panel A, illustrates health care spending by type of care, sex, and aggregated condition category

Supplemental Figure 4: Health Care Spending in Norway by Type of Care and Age, 2019

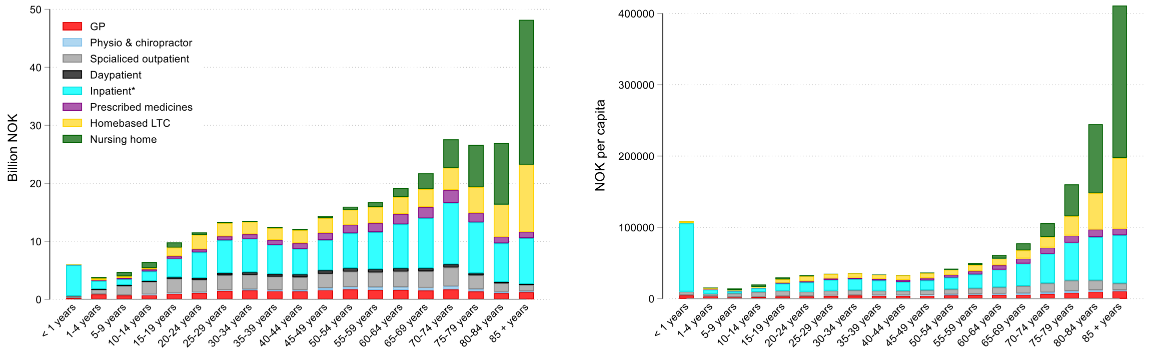

Panel A, illustrates health care spending by type of care and age. Panel B, illustrates health care spending per capita by type of care and age. Increases in spending along the y-axis show more spending.

Supplemental Figure 5: Top 10 Causes of Spending by Type of Care and Sex, 2019

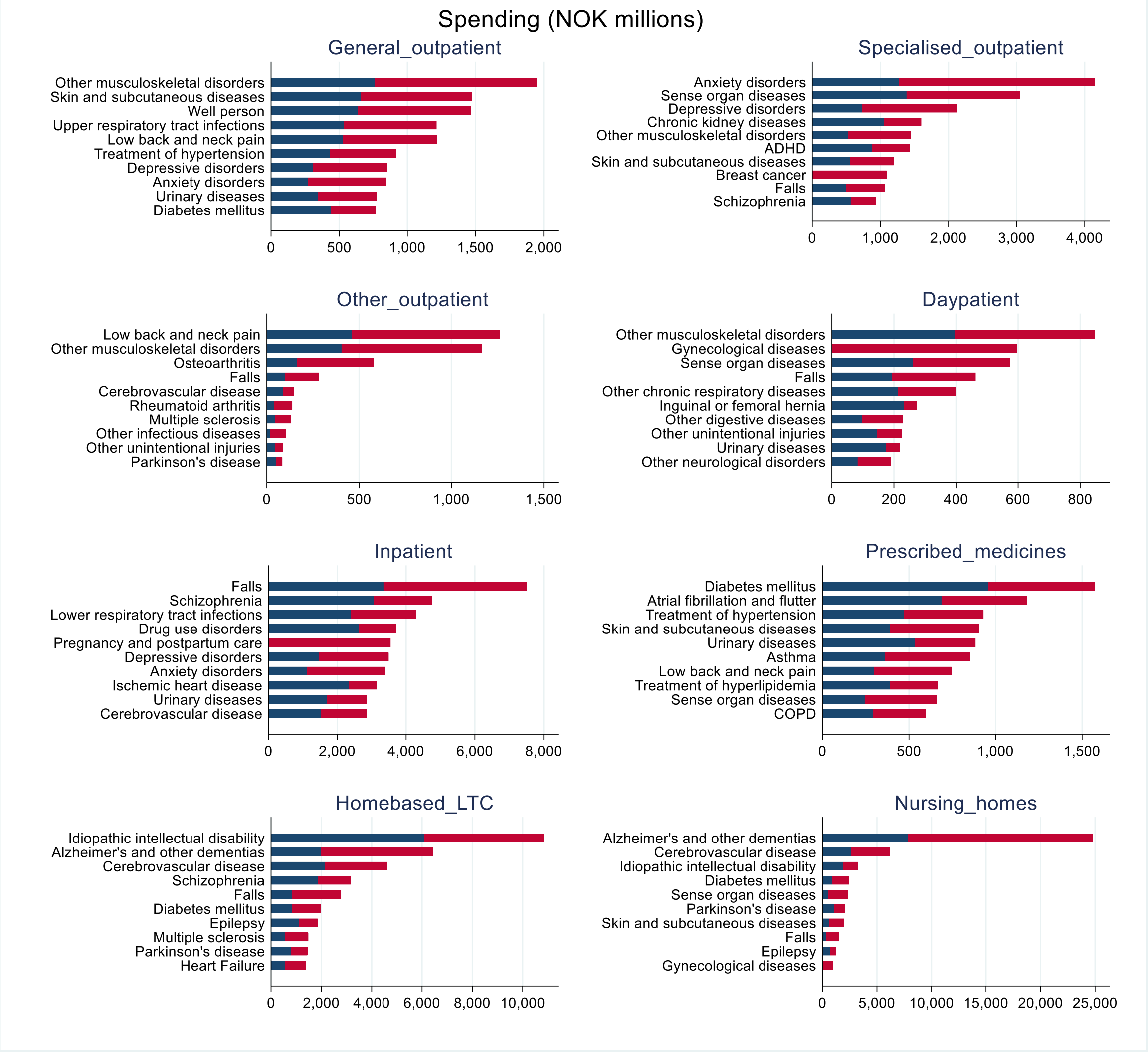

References

1. The Norwegain Directorate of Health: SAMDATA spesialisthelsetjeneste 2019 [in Norwegian]. The Norwegain Directorate of Health,; 2020.
2. Mjåset C, Byrkjeflot H, Hanssen F, Wynn-Jones W: An introduction to bundled payments. Tidsskr Nor Laegeforen 2020, 140(12).
3. Bjørnelv GM, Halsteinli V, Kulseng BE, Sonntag D, Ødegaard RA: Modeling Obesity in Norway (The MOON Study): A Decision-Analytic Approach—Prevalence, Costs, and Years of Life Lost. Medical Decision Making 2021, 41(1):21-36.
4. The Norwegain Directorate of Health: Kostnadsnivå og produktivitet i psykisk helsevern og TSB 2014-2018 [in Norwegian]. In. Oslo: The Norwegain Directorate of Health; 2019.
5. Bakken IJ, Ariansen AM, Knudsen GP, Johansen KI, Vollset SE: The Norwegian Patient Registry and the Norwegian Registry for Primary Health Care: Research potential of two nationwide health-care registries. Scandinavian journal of public health 2020, 48(1):49-55.
6. The Norwegain Directorate of Health: The KUHR-database [in Norwegian]. In.: The Norwegain Directorate of Health,; 2004.
7. The Norwegian Directorate of Health: Økonomisk evaluering av helsetiltak – en veileder [in Norwegian]. Oslo: The Norwegian Directorate of Health,; 2012.
8. Brathaug AL: Conversation about services included in hombased care in the National Health Accounts; 2021.
9. Dieleman JL, Cao J, Chapin A, Chen C, Li Z, Liu A, Horst C, Kaldjian A, Matyasz T, Scott KW: US health care spending by payer and health condition, 1996-2016. Jama 2020, 323(9):863-884.
10. Wieser S, Riguzzi M, Pletscher M, Huber CA, Telser H, Schwenkglenks M: How much does the treatment of each major disease cost? A decomposition of Swiss National Health Accounts. The European Journal of Health Economics 2018, 19(8):1149-1161.
11. Statistics Norway: National accounts - Health accounts. Statistics Norway; 2021.
12. Rosen AB, Cutler DM: Challenges in building disease-based national health accounts. Med Care 2009, 47(7 Suppl 1):S7-13.
13. Sælensminde K, Line T: Samfunnskostnader ved sykdom og ulykker 2015. Helsedirektoratet (IS-2839 Rapport) Hentet fra: https://www.helsedirektoratet.no/rapporter/samfunnskostnader-ved-sykdom-ogulykker/Samfunnskostnader%20ved%20sykdom%20og%20ulykker 2019, 2020.
14. Published. WHOJ: International Classification of Primary Care (ICPC-2) Disponible en: http://www.who.int/classifications/icd/adaptations/icpc2/en. 2016.
15. World Health Organization: International Classification of Primary Care (ICPC-2) In.: World Health Organization; 2016.
16. Holm SG, Mathisen TA, Sæterstrand TM, Brinchmann BS: Allocation of home care services by municipalities in Norway: a document analysis. BMC Health Services Research 2017, 17(1):673.
17. Mueller M, Bourke E, Morgan D: Assessing the comparability of long-term care spending estimates under the Joint Health Accounts questionnaire. Paris: OECD; 2020.
18. Hernæs KH, Brathaug AL: Helseregnskapet–et internasjonalt system. Økonomiske analyser 2012, 2(2012):18-23.
19. KOSTRA [https://www.ssb.no/en/offentlig-sektor/kostra/statistikk/kostra-kommune-stat-rapportering]
20. Care services [https://www.ssb.no/en/helse/helsetjenester/statistikk/sjukeheimar-heimetenester-og-andre-omsorgstenester]
